# AI-based image quality assessment of positioning in mammography: considerations and challenges

**DOI:** 10.1186/s13244-025-02191-3

**Published:** 2026-02-16

**Authors:** Tina Santner, Mickael Tardy, Johanne-Gro Stalheim, Stephanie Frei, Wolfram Santner, Stefano Gianolini, Malik Galijasevic, Marthe Larsen, Jonas Gjesvik, Solveig Hofvind, Gerlig Widmann

**Affiliations:** 1 Medical University of Innsbruck, Innsbruck, Austria; 2 Hera-MI, Saint-Herblain, France; 3 Evidia, Bergen, Norway; 4 Way to Women Sàrl, Le Vaud, Switzerland; 5 Team Radiologie Plus, Chur, Switzerland; 6 MSS Medical Software Solutions GmbH, Erlenbach, Switzerland; 7 Medical University of Innsbruck, Department of Radiology, Innsbruck, Austria; 8 Cancer Registry of Norway, Norwegian Institute of Public Health, Oslo, Norway

**Keywords:** Mammography, Quality, PGMI, Software, AI

## Abstract

**Objectives:**

Artificial intelligence (AI) could facilitate and objectify quality assessment in the daily routine. The purpose was to explore the extent to which an AI prototype algorithm is able to replicate the perfect-good-moderate-inadequate (PGMI) system (perfect, good, moderate, inadequate).

**Materials and methods:**

From a multicentre case collection, 200 standard mammograms (800 images) were selected. A deep learning-based prototype software was used to rate the images in analogy to the PGMI system. The AI results were compared with a reference standard obtained through consensus reading by three expert radiographers and one expert radiologist, using quadratically weighted Cohen’s kappa with confidence intervals (CI) and context-based interpretation. Frequency and reasons for disagreement were evaluated for challenging cases with a discrepancy of two or more grades and a discrepancy in assigning an inadequate.

**Results:**

For overall PGMI per image, slight agreement between human consensus and AI was observed for CC views (κ = 0.14) and fair agreement for MLO views (κ = 0.25). The highest agreement was observed for the CC category “M. Pectoralis visibility” (substantial, κ = 0.75). Best category in MLO was “Pectoralis angle” (moderate, κ = 0.49). For other categories, fair, slight or poor agreement was observed. The work-up of disagreement gave insight into misinterpretations of anatomical landmarks and causality issues in the categorization.

**Conclusion:**

Transforming the PGMI system into a fully automated AI algorithm is challenging and may differ substantially between subcategories. Further research in computer science and quality assessment methodology is needed to pave the way for AI-based objective quality management in mammography.

**Critical relevance statement:**

Profound evaluation of AI algorithms and their ability to replicate human interpretation, scoring, and classification are the basis and scientific framework toward AI-based objective quality management in mammography.

**Key Points:**

- AI has huge potential for automated assessment of diagnostic image quality.
- Compared with human reading agreement, substantial disagreement may also be found.
- Direct transformation of perfect-good-moderate-inadequate scoring into an AI algorithm is challenging.

**Graphical Abstract:**

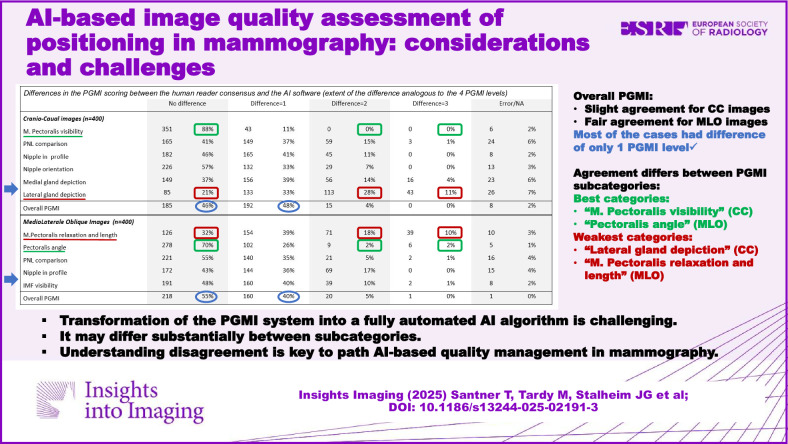

## Introduction

Breast cancer is the most commonly diagnosed cancer type and cause of cancer deaths among women worldwide [1]. Mammography is considered the best screening tool for the disease, and about 40 million examinations are performed each year in the United States alone [2]. High-quality images are a prerequisite for high sensitivity and specificity of mammographic screening [3]. Correct positioning of the breast is essential to fully visualize and correctly display all relevant tissue to avoid misinterpretations of features or missing tumors [4, 5]. As radiographers make autonomous decisions throughout the process and in their interaction with women, awareness of their responsibility and reflection on their own performance should be encouraged [6]. Various classification and grading systems have been developed to determine the image quality of a mammogram [7]. The Perfect-Good-Moderate-Inadequate (PGMI) system, originally conceived for mammography screening by the National Health Service in the United Kingdom (NHSBSP), has been used and adapted internationally for decades [3, 7–10]. It corresponds to a catalog of criteria, which enables a systematic analysis of each view, craniocaudal (CC) and mediolateral oblique (MLO), resulting in an overall image quality score. Available guidelines from different countries state that ≥ 75% of the screening images should reach a P or G, ≤ 22% M, and ≤ 3% I [3, 11]. However, despite the given rules and recommendations, interpretation remains subjective and is prone to inter- and intrareader variability [7, 12]. Hofvind et al [3] observed that the distributions of given PGMI grades differed significantly between local readers and a superior expert. Hill/Robinson [6] analyzed the vague wording and misleading definitions of guidelines and concluded the tool to be neither reliable nor valid. Boyce et al [9] also addressed these ambiguities and found poor agreement between results evaluated by assessors from different countries. Alukic et al [13] measured mainly poor agreement between evaluations of five radiographers, with subjectivity being a major influence despite clearly communicated rules. Furthermore, an organized evaluation of image quality, and dissemination of results and improvement actions requires resources and is time-consuming [7, 14]. Consequently, randomly selected images are usually assessed for each radiographer; they may not be fully representative and may not capture all complex cases. Efforts should thus be made to find a practical method with high efficiency that gives comprehensive support to the radiographers and fits into their daily routine [6].

Use of artificial intelligence (AI) generally opens opportunities in facilitating and accelerating working processes and might also help to simplify and automate aspects of image quality assurance [2, 12, 13]. First prototypes of software solutions for quality evaluation of mammograms have been introduced, which may provide live feedback during the examination or report quality assessment and monitoring data retrospectively [12, 14]. These systems might be able to replace subjective human measurements, perform faster, be more comprehensive, and identify training needs distinctively to enable interventions [12]. However, there is limited knowledge about the performance of these systems and how they can replicate human interpretations, scorings and classifications. The aim of the study was to evaluate whether an AI algorithm matches human reference PGMI scores from clinical routine data. Further, we wanted to understand and describe the reasons for the major discrepancy.

## Materials and methods

The study was approved by the ethical committee (Medical University of Innsbruck, Austria, reference number 1321/2021), which waived the requirement for informed patient consent.

### Study population

We received image data from 200 anonymized standard digital mammography examinations from 13 sites in university hospitals and private clinics, 100 from women residing in Switzerland, and 100 from women residing in Austria. The mammograms were performed in the period from June to August 2021 with mammography machines from three vendors (GE *n* = 26; Hologic *n* = 52 and Siemens *n* = 122). All women had four images, two CC and two MLO, resulting in 800 images in total.

### Human PGMI reference and AI-based evaluation of image quality

The Austrian version of the PGMI system [11] was used and slightly refined to avoid presumably vague definitions ([6, 9], Appendix 1). The analysis included 16 criteria for CC and 15 criteria for MLO (Appendix 1). In addition, a summary PGMI score was provided for each image. All 800 images were independently classified by three PGMI expert radiographers with more than 10 years of experience using PGMI in Austria (T.S.), Norway (J.S., T.S.), or Switzerland (S.F., T.S.). In any reading process, all image parameters were hidden. To establish a human reference standard, the three independent assessments were reviewed and consolidated by a radiographer (T.S.) and a breast radiologist (W.S.), both with over 10 years of experience in breast imaging from Austria, Norway, and Switzerland. For each of the 800 images, T.S. and W.S. manually examined the ratings across all individual positioning criteria for both CC and MLO views. A consensus decision was reached for each criterion, and these were then used to derive a final PGMI score per image. In cases of disagreement or ambiguity among the three original ratings, T.S. and W.S. jointly reviewed the image and resolved differences through consensus-based adjudication. T.S. and W.S.’s intervention was not to judge or even overrule the decision of the three readers, but solely to resolve cases that could not produce a consensus result. They were blinded to the AI outputs when performing the adjudication. Such a double-review approach ensured consistent scoring and provided an arbitration mechanism when majority voting alone was insufficient.

The 800 images were classified as either challenging or non-challenging based on predefined criteria for challenging cases. An image was considered challenging if any of the following conditions applied: (1) accurate measurement of the pectoralis nipple line (PNL) and its deviation was not possible due to a short pectoralis muscle on the MLO view combined with its absence on the CC view; (2) the pectoralis muscle and/or nipple appeared frayed or blurred, hindering precise PNL measurement; (3) the nipple was not clearly visible due to anatomical, positioning, or technical factors (e.g., inadequate compression, suboptimal exposure, image noise, or poor contrast); or (4) differentiation between skinfolds and scars was difficult.

The research prototype software (Hera-MI Mammography Technical Evaluation) was based on an existing CE-certified product (Breast SlimView), including developments for quality assessment (Mammography Technical Evaluation). The specific algorithm relied on a deep neural network which was trained in a supervised approach on a large set of multivendor data independent from the present study (seven institutions in Europe; training 12,000 images, validation 2400, testing 1200; Fujifilm (26%), Hologic (22%), GE (17%), IMS Giotto (10%), Siemens (10%), Planmed (7%)) in a multi-task manner, based on annotations generated by three professionals with 1–5 years of experience in mammography who were not the readers of this study and unknown to this group, similarly described in Tardy et al [15]. The following classification tasks were performed by the software: nipple correctness, IMF correctness, pectoral muscle presence. The following objects were segmented: pectoral muscle, nipple, IMF and glandular tissue. The assessment of the correctness of nipple and IMF was used from the network output, same is for glandular tissue. Other outputs, the angle and length measurements, such as PNL, pectoral muscle angle, were calculated from the segmentation results and were not obtained directly by training on PGMI-labeled datasets. This limits the fully AI mode of operations and contributes to the explainability of the algorithm.

Preparation and validation of the algorithm were conducted by the AI provider; no additional training was performed during the study. The evaluation of the algorithm relied on the ground truth generated by skilled professionals as part of the research and development process of the manufacturer. Each model component was validated independently; overall PGMI-mapping performance was evaluated using a separate PGMI-labeled dataset. To verify that angle and length outputs met the accuracy requirements for clinical quality control, mean absolute errors (MAE) were computed. MAE for pectoral angle was below 2 degrees, while MAE of PNL was below 2 mm.

A separate, deterministic rule layer assigns the information from all measurements to a scale that reflects the level graduated from the ideal image. In the software, this was translated into grades on a scale between 0 and 10. The conversion into PGMI categories required for the study was defined as follows: I = [0, 2], M = [3, 5], G = [6, 9], P = 10. Thus, the AI performs detection/measurement; the logical layer implements transparent, rule‑based stratification.

The visible output of the software would include a report per study with a global grade and a list of deficiencies per image (Appendix 2).

For the study, PGMI criteria and rules were matched as close as possible to the scheme used by the human readers.

The software version used in this study focused on positioning criteria. Skinfold and blur/motion detection were not implemented, as these features were outside the scope of the current release and scheduled for future iterations. We discuss implications for benchmarking against expert readers in the “Limitations” section. Appendix 1 provides an overview of which criteria were assessed by the human readers and also processed by the software, as well as which of the scored criteria were included in the statistical analysis of the study.

The software results for the 800 images were entered into the predesigned CRF for comparison with the human consensus reading.

### Statistical analysis and review

We descriptively presented the distribution of PGMI scores by frequencies and percentages, for the human consensus and for the AI system. The results were stratified by CC and MLO images. The results were further stratified by non-challenging and challenging cases.

The agreement between the human consensus and the AI system for each PGMI category was summarized using quadratically weighted Cohen’s kappa [16], including the corresponding confidence intervals (CIs). Agreement was also presented for the overall PGMI value in CC and MLO, supplemented by confusion matrices. The strength of agreement was interpreted in a context-dependent manner, as the Landis and Koch thresholds (< 0 poor agreement, 0–0.20 slight agreement, 0.21–0.40 fair agreement, 0.41–0.60 moderate agreement, 0.61–0.80 substantial agreement, > 0.81 almost perfect agreement) [17] strictly apply only to unweighted Cohen’s kappa.

Statistics were calculated in Stata (StataCorp. 2023. Stata Statistical Software: Release 19. College Station, TX: StataCorp LLC) using the kappaetc package (Kolenikov [18]), R Statistical Software (R Core Team v. 3.3.0), and Microsoft Excel Version 2409.

All images in the challenging group, in which the overall PGMI between the human consensus and the AI differed by two or three levels, or in which there was disagreement about inadequacy, were reviewed by an expert radiographer (T.S.). To identify the reason for deviation, the content of the CRFs was manually compared value by value and linked to the respective image. Findings for discrepancy between the human readers and the AI system were described qualitatively (expert radiographer review).

## Results

### Consensus intervention

The three readers agreed in 156 (30%) of the non-challenging cases and 90 (32.14%) of the challenging cases. A decision was produced by majority voting in 347 (66.73%) and 168 (60%) cases, respectively. In 13 cases (2.5%), respectively 17 cases (6.07%), an adjudication by T.S./W.S. was necessary. In 4 (0.76%) and 5 (1.79%) of the cases, respectively, no reliable interpretation was possible due to unclear landmarks in the images.

### Descriptives

**Fig. 1:**
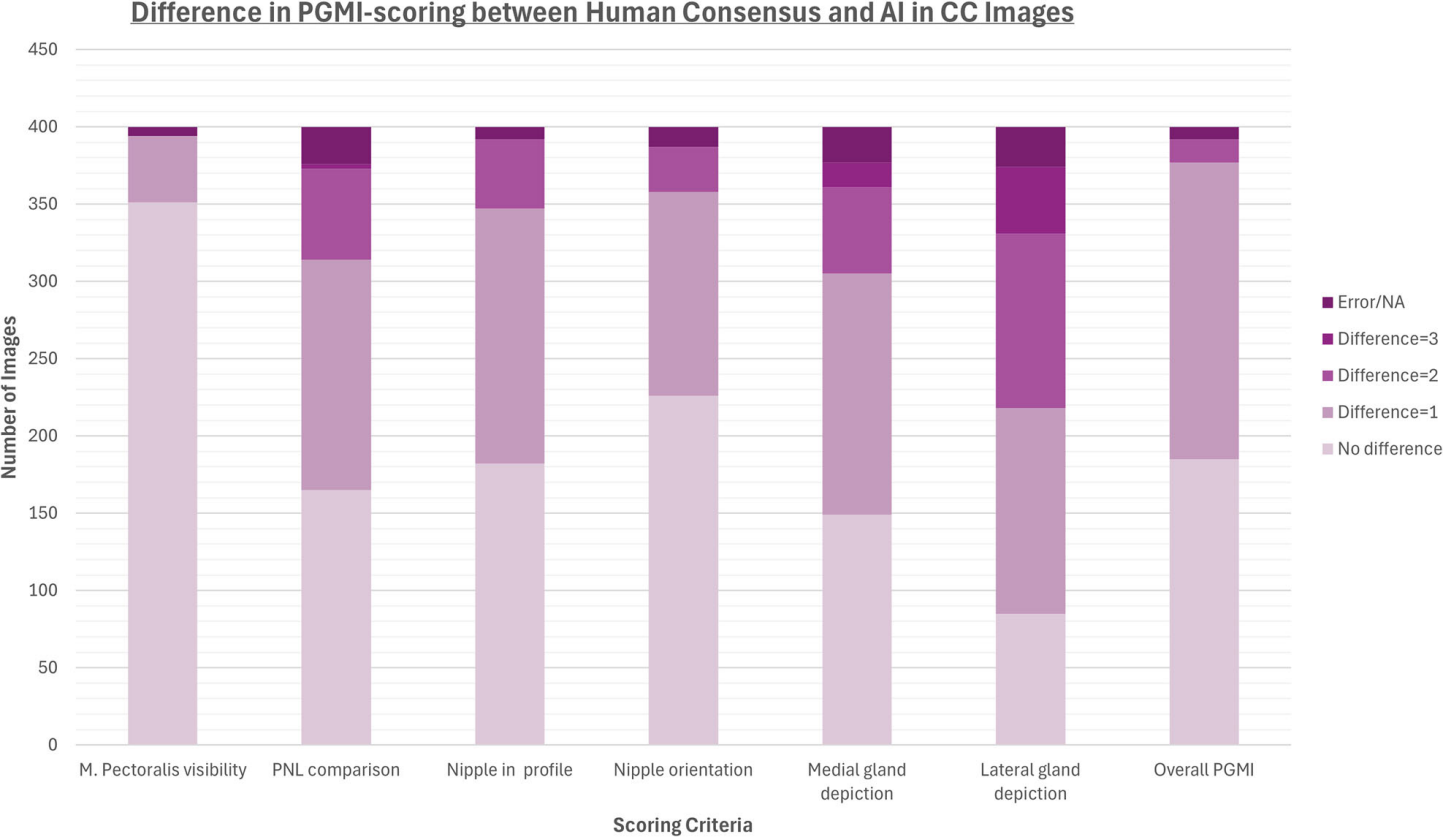
Differences in the PGMI (Perfect, Good, Moderate, Inadequate) scoring between the human consensus and the AI in CC images. Error/NA referred to no consensus possible, or too little information on the image to give a fair grade

**Fig. 2:**
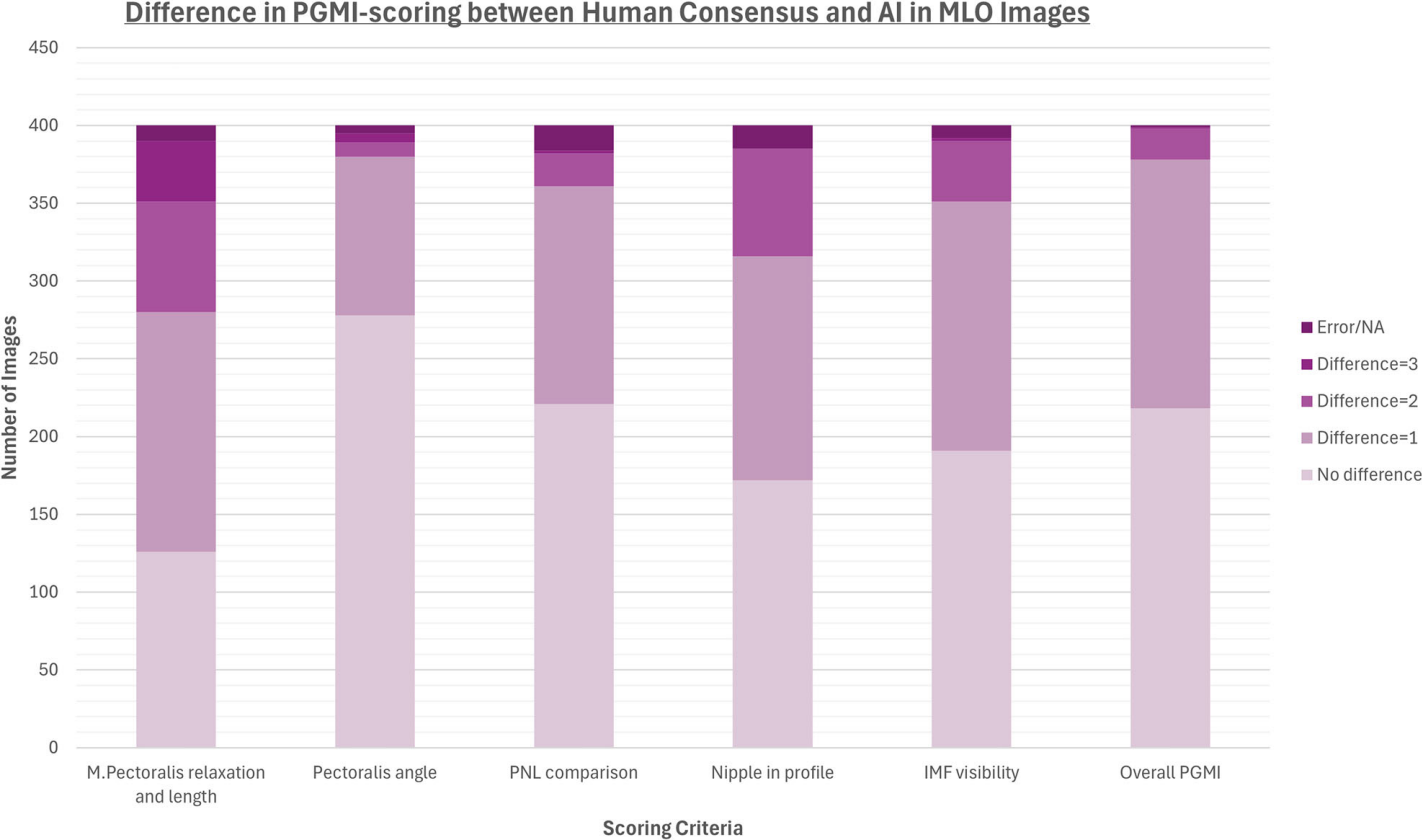
Differences in the PGMI (Perfect, Good, Moderate, Inadequate) scoring between the human consensus and the AI in MLO images. Error/NA referred to no consensus possible, or too little information on the image to give a fair grade

Frequencies and percentages of perfect, good, moderate, and inadequate scores for each PGMI category for the 400 CC images and 400 MLO images, stratified by human reference and artificial intelligence, are depicted in Table 1. For CC images, no difference in classification between the human reference and AI was observed in 88% (351/400) for the category “M. Pectoralis visibility,” 57% (226/400) for “Nipple orientation,” and 46% (182/400) for “Nipple in profile” (Fig. 1, Table 2). For “Lateral gland depiction,” 11% (43/400) had a difference of 3 levels between human reference and AI. In MLO, no difference in the PGMI classification between human reference and AI was observed in 70% (278/400) for the category “Pectoralis angle,” followed by 55% (221/400) for “PNL comparison” and 48% (191/400) for “IMF visibility” (Fig. 2, Table 2). A difference of 2 and 3 levels was observed in 18% (71/400) and 10% (39/400) of the category “M. Pectoralis relaxation and length,” respectively, and a difference of 2 levels was observed in 17% (69/400) of the category “Nipple in profile.” For the overall PGMI, a difference of 2 or more levels was observed in 5% (15/400) for CC and 6% (21/400) for MLO, respectively.

**Table 1:** Frequencies and percentages of P, G, M, and I (perfect, good, moderate, inadequate) scores for each PGMI category for 400 CC images and 400 MLO images

	Human readers										Artificial Intelligence							
	Perfect		Good		Moderate		Inadequate		Error/NA		Perfect		Good		Moderate		Inadequate	
	*n*	%	*n*	%	*n*	%	*n*	%	*n*	%	*n*	%	*n*	%	*n*	%	*n*	%
Craniocaudal images (*n* = 400)																		
M. Pectoralis visibility	138	35%	256	64%	0	0%	0	0%	6	2%	119	30%	281	70%	0	0%	0	0%
Pectoralis-nipple-line comparison	124	31%	110	28%	117	29%	25	6%	24	6%	205	51%	147	37%	48	12%	0	0%
Nipple in profile	291	73%	60	15%	24	6%	17	4%	8	2%	159	40%	164	41%	77	19%	0	0%
Nipple orientation	191	48%	114	29%	70	18%	12	3%	13	3%	299	75%	83	21%	18	5%	0	0%
Medial gland depiction	149	37%	156	39%	56	14%	16	4%	23	6%	400	100%	0	0%	0	0%	0	0%
Lateral gland depiction	91	23%	114	29%	107	27%	62	16%	26	7%	365	91%	0	0%	35	9%	0	0%
Skinfolds	165	41%	183	46%	43	11%	2	1%	7	2%	n.a.	n.a.	n.a.	n.a.	n.a.	n.a.	n.a.	n.a.
Overall PGMI	20	5%	166	42%	159	40%	47	12%	8	2%	38	10%	228	57%	134	34%	0	0%
Mediolateral oblique images (*n* = 400)																		
M. Pectoralis shape and length	111	28%	151	38%	78	20%	50	13%	10	3%	345	86%	27	7%	28	7%	0	0%
M. Pectoralis angle	147	37%	198	50%	38	10%	12	3%	5	1%	102	26%	249	62%	23	6%	26	7%
Pectoralis-nipple-line comparison	292	73%	66	17%	18	5%	8	2%	16	4%	235	59%	139	35%	26	7%	0	0%
Nipple in profile	230	58%	86	22%	42	11%	27	7%	15	4%	132	33%	106	27%	162	41%	0	0%
Inframammary fold visibility	159	40%	118	30%	66	17%	49	12%	8	2%	211	53%	138	35%	51	13%	0	0%
Skinfolds	102	26%	209	52%	78	20%	11	3%	0	0%	n.a.	n.a.	n.a.	n.a.	n.a.	n.a.	n.a.	n.a.
Overall PGMI	16	4%	120	30%	203	51%	60	15%	1	0%	13	3%	150	38%	211	53%	26	7%

Results are stratified by human consensus and artificial intelligence (AI). Error/NA referred to no consensus possible or not sufficient information on the image to give a fair grade

**Table 2:** Differences in the PGMI (Perfect, Good, Moderate, Inadequate) scoring between the human consensus and the AI

	No difference		Difference = 1		Difference = 2		Difference = 3		Error/NA	
Craniocaudal images (*n* = 400)										
M. Pectoralis visibility	351	88%	43	11%	0	0%	0	0%	6	2%
PNL comparison	165	41%	149	37%	59	15%	3	1%	24	6%
Nipple in profile	182	46%	165	41%	45	11%	0	0%	8	2%
Nipple orientation	226	57%	132	33%	29	7%	0	0%	13	3%
Medial gland depiction	149	37%	156	39%	56	14%	16	4%	23	6%
Lateral gland depiction	85	21%	133	33%	113	28%	43	11%	26	7%
Overall PGMI	185	46%	192	48%	15	4%	0	0%	8	2%
Mediolateral oblique images (*n* = 400)										
M. Pectoralis relaxation and length	126	32%	154	39%	71	18%	39	10%	10	3%
Pectoralis angle	278	70%	102	26%	9	2%	6	2%	5	1%
PNL comparison	221	55%	140	35%	21	5%	2	1%	16	4%
Nipple in profile	172	43%	144	36%	69	17%	0	0%	15	4%
IMF visibility	191	48%	160	40%	39	10%	2	1%	8	2%
Overall PGMI	218	55%	160	40%	20	5%	1	0%	1	0%

A difference of 1 could, for example, mean that AI scored an image as P and human consensus as G. If the difference was 3, AI had scored P and human consensus I, or the other way around. Error/NA referred to no consensus possible or too little information on the image to give a fair grade. Results stratified by non-challenging and challenging cases are given in Appendix 3

For CC images, the highest agreement between the human reference and the AI system was observed for “M. pectoralis visibility” (κ = 0.75) (Table 3). Medium agreement was observed for overall PGMI for CC images (κ = 0.41). Considering the MLO images, the strongest agreement (κ = 0.57) between human consensus and AI was observed for “Pectoralis angle.” Agreement for the overall PGMI rating was slightly lower in MLO compared with CC (κ = 0.38). Across the remaining categories in both CC and MLO views, agreement tended to be lower.

**Table 3:** Quadratically weighted Cohen’s kappa and 95% confidence intervals (CI) between human consensus and the AI for craniocaudal (CC) and mediolateral oblique (MLO) images for each PGMI category (Perfect, Good, Moderate, Inadequate), and for all, non-challenging and challenging cases

	All cases, *n* = 400		Non-challenging, *n* = 260		Challenging, *n* = 140	
Craniocaudal images (CC)	*n*	Kappa (95% CI)	*n*	Kappa (95% CI)	*n*	Kappa (95% CI)
M. Pectoralis visibility	394	0.75 (0.68–0.82)	255	0.74 (0.65–0.83)	139	0.77 (0.66–0.89)
PNL comparison	376	0.33 (0.25–0.41)	245	0.47 (0.38–0.56)	131	0.11 (−0.02 to 0.23)
Nipple in profile	392	0.33 (0.25–0.41)	257	0.48 (0.37–0.60)	135	0.08 (−0.01 to 0.17)
Nipple orientation	387	0.48 (0.40–0.56)	252	0.46 (0.36–0.56)	135	0.47 (0.35–0.59)
Medial gland depiction	377	0.00 (0.00–0.00)	247	0.00 (−0.00 to 0.00)	130	−0.00 (−0.00 to −0.00)
Lateral gland depiction	374	0.08 (0.02–0.13)	243	0.00 (−0.06 to 0.07)	131	0.13 (0.05–0.20)
Overall PGMI	392	0.41 (0.34–0.47)	256	0.37 (0.34–0.50)	136	0.30 (0.18–0.42)
Mediolateral oblique images (MLO)						
M. Pectoralis relaxation and length	390	0.08 (0.03–0.14)	253	0.09 (0.01–0.17)	137	0.08 (0.02–0.15)
Pectoralis angle	395	0.57 (0.47–0.67)	259	0.59 (0.47–0.71)	136	0.53 (0.35–0.71)
PNL comparison	384	0.26 (0.15–0.37)	252	0.39 (0.25–0.53)	132	0.06 (−0.12 to 0.23)
Nipple in profile	385	0.39 (0.31–0.46)	250	0.63 (0.55–0.71)	135	−0.00 (−0.06 to 0.05)
IMF visibility	392	0.49 (0.45–0.57)	260	0.52 (0.43–0.62)	132	0.46 (0.36–0.55)
Overall PGMI	399	0.38 (0.29–0.47)	260	0.36 (0.25–0.48)	139	0.12 (−0.01 to 0.26)

The number of images included in the PGMI analysis for each category is also provided

Confusion matrices with the number of images classified as P, G, M and I by AI and human consensus in the overall PGMI can be seen in Table 4.

**Table 4:** Confusion matrices showing frequencies classified as P, G, M, and I (Perfect, Good, Moderate, Inadequate) by artificial intelligence (AI) and human consensus for the overall PGMI of craniocaudal (CC) and mediolateral oblique (MLO) images, for all cases, non-challenging cases, and challenging cases

		CC images Human consensus				MLO images Human consensus			
	All cases *n* = 392 for CC; *n* = 399 for MLO	P	G	M	I	P	G	M	I
AI	P	8	28	2	0	3	5	4	1
	G	11	110	90	12	7	74	64	4
	M	1	28	67	35	6	35	128	42
	I	0	0	0	0	0	6	7	13
	Non-challenging cases *n* = 256 for CC; *n* = 260 for MLO	P	G	M	I	P	G	M	I
AI	P	8	28	2	0	3	5	4	1
	G	7	77	65	7	7	72	62	3
	M	0	11	38	13	2	14	6	13
	I	0	0	0	0	0	4	4	6
	Challenging cases *n* = 136 for CC; *n* = 139 for MLO	P	G	M	I	P	G	M	I
AI	P	0	0	0	0	0	0	0	0
	G	4	33	25	5	0	2	2	1
	M	1	17	29	22	4	21	68	29
	I	0	0	0	0	0	2	3	7

For non-challenging images, fair agreement was observed in the overall PGMI in both CC and MLO (κ = 0.37 and κ = 0.36). For challenging images, agreement was reduced to varying degrees, being moderately lower in CC (κ = 0.30) and markedly lower in MLO (κ = 0.12).

The challenging images had minor agreement in the categories “PNL comparison” and “Nipple in profile” in both CC and MLO views. Details can be seen in Table 3.

We found a difference of 3 levels for “Medial gland depiction” (5%, 12/260) and “Lateral gland depiction” (7%, 17/260) for non-challenging CC cases (Appendix 3). It was 3% (4/140) for “Medial gland depiction” and 19% (26/140) for “Lateral gland depiction” for the challenging cases. A difference of 3 levels was found in 6% (15/260) of the “M. Pectoralis relaxation and length” for the non-challenging cases and in 17% (24/140) of the challenging cases.

### Reasons for the discrepancy between human reference and AI reading

**Fig. 3:**
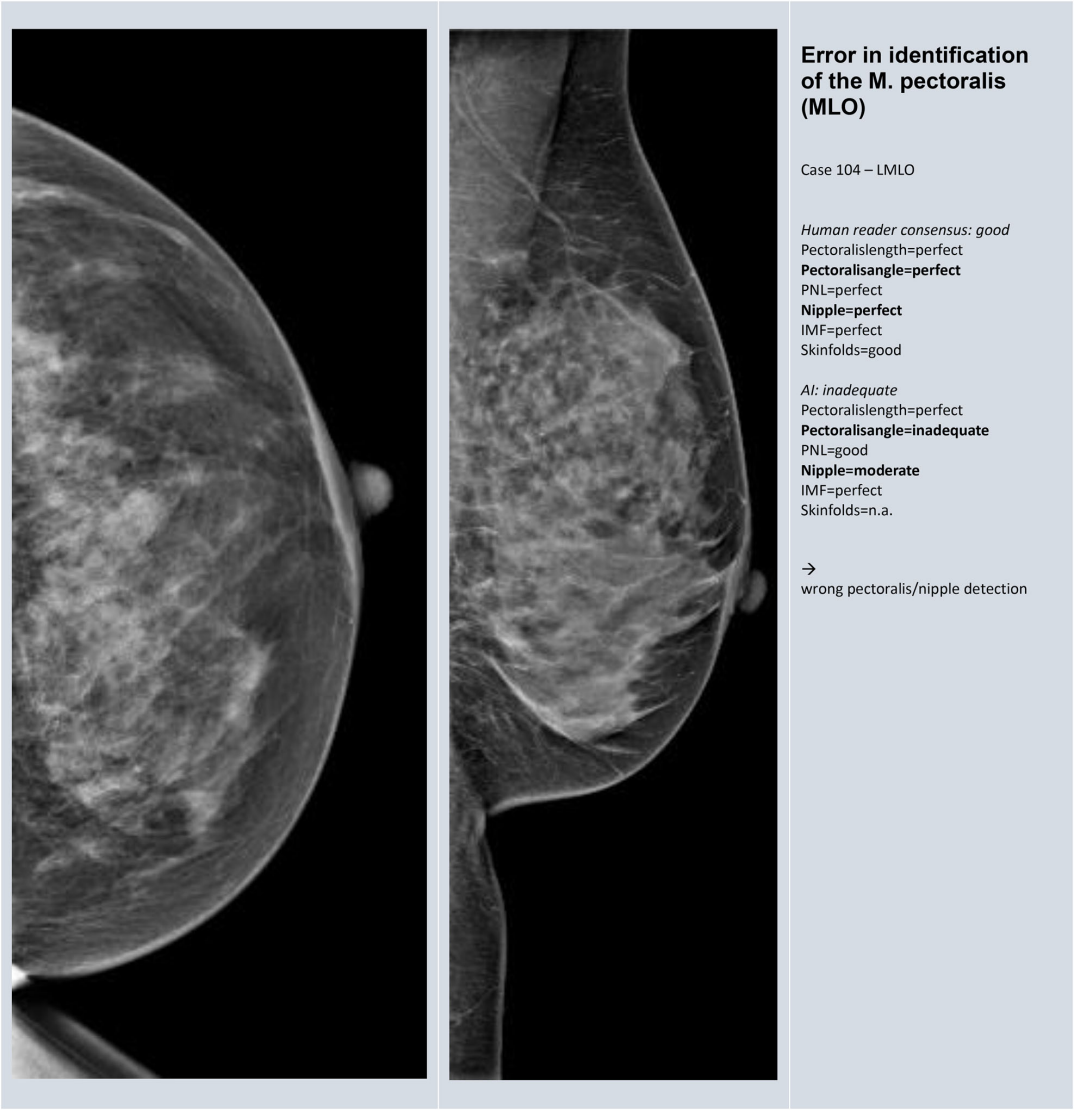
Example for discrepancy in the identification of the M. pectoralis in MLO view

**Fig. 4:**
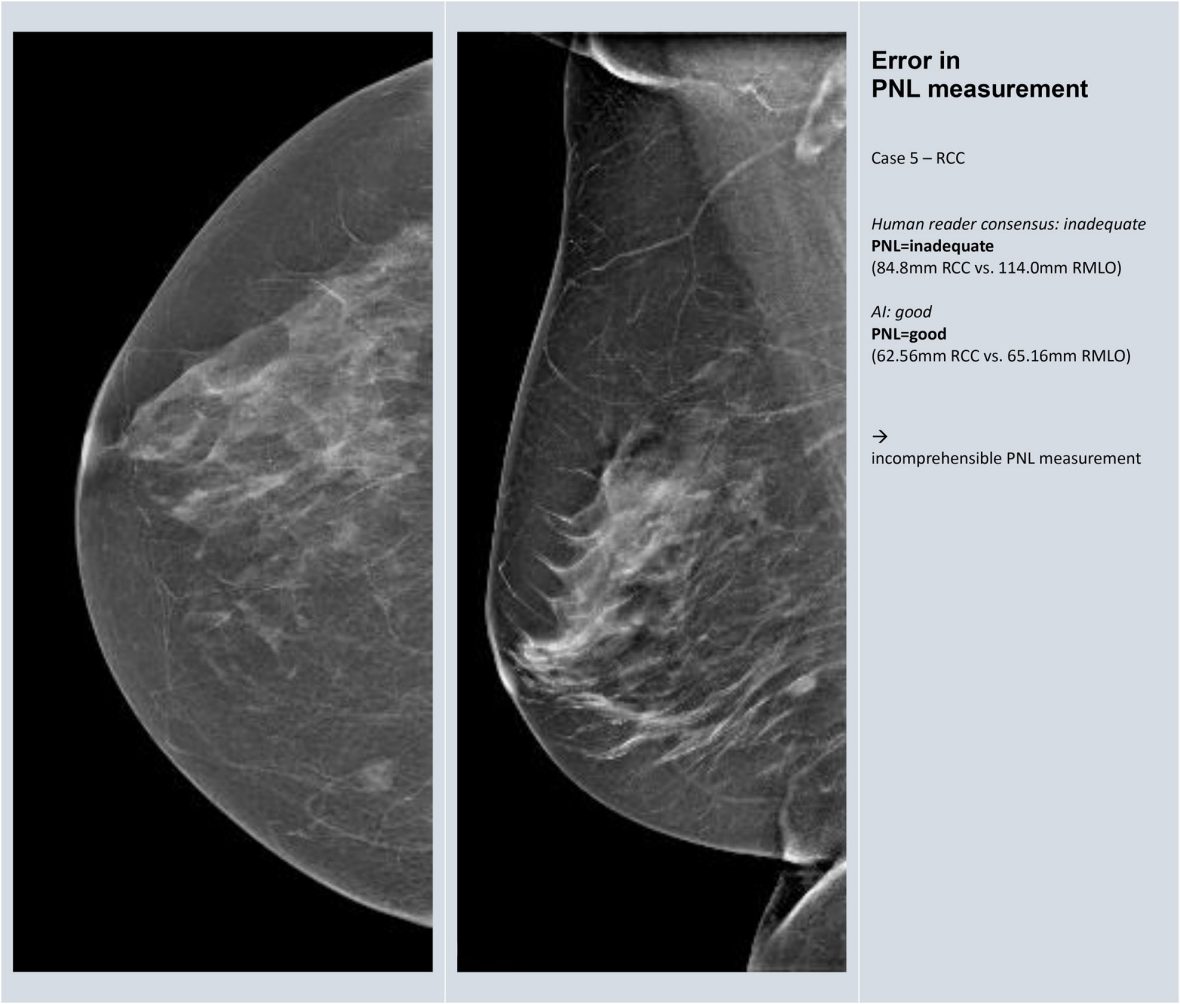
Example for discrepancy in PNL measurements

**Fig. 5:**
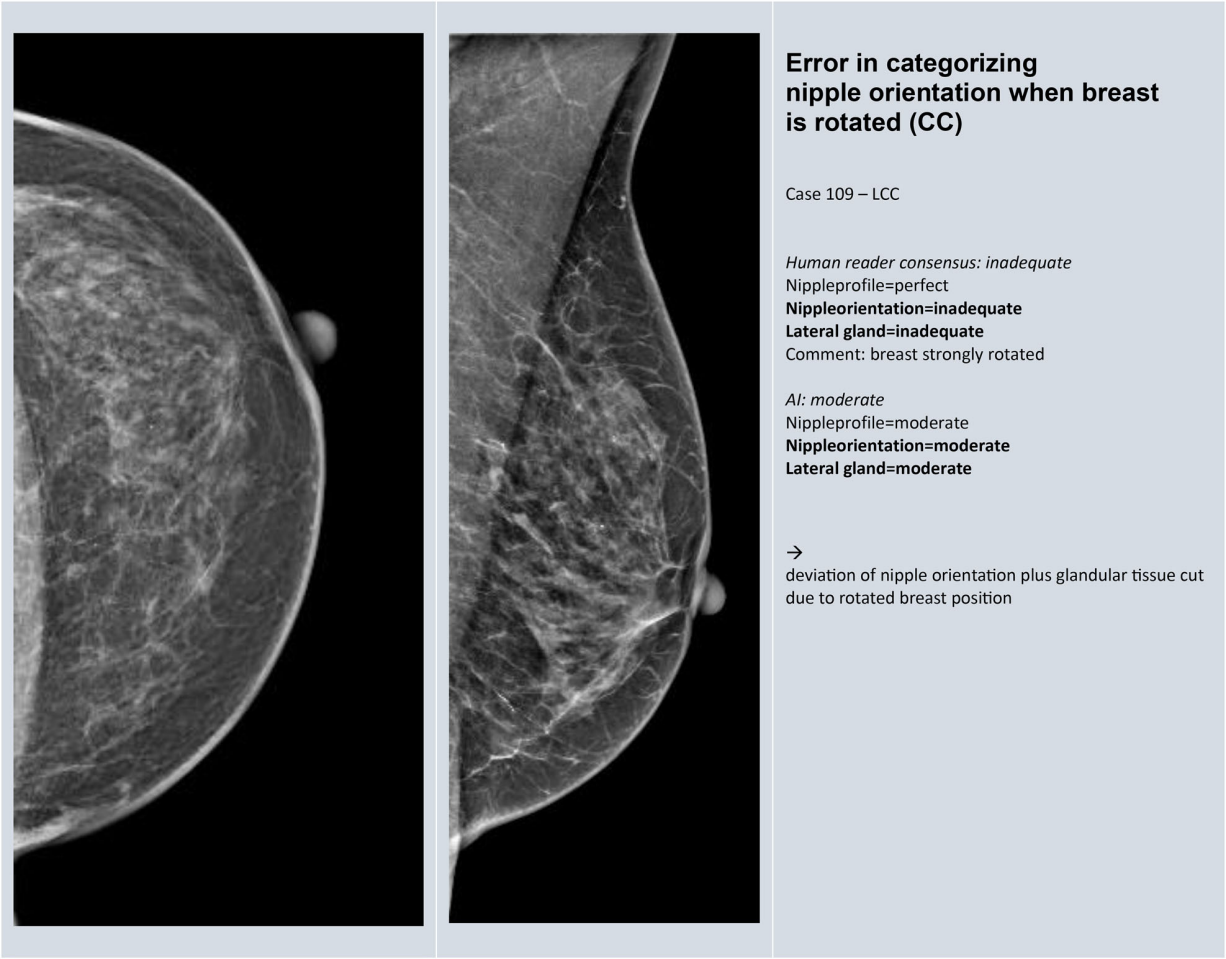
Example for discrepancy in categorizing the nipple orientation when the breast is rotated in CC view

A total of 75 images (33 CC and 42 MLO) from the challenging group met the conditions for further evaluation to identify reasons for discrepancy. The reasons and frequency of discrepancy between human reference and AI reading are given in Table 5. In 17 (23%) images, M. Pectoralis was incorrectly identified in MLO (Fig. 3). Such cases were identified throughout, independent of the image contrast and postprocessing of different vendors. In 19 (25%) of the images, the PNL measurement was not readily comprehensible by the AI, and the landmarks upon which the measurement was based remained unclear (Fig. 4). Uncertainty as to whether the nipple was correctly registered and classified by the AI was a reason for discrepancy between human consensus and AI in the same number of cases. In 12 (16%) CC images, the orientation of the nipple was deviated while the lateral glandular tissue was simultaneously cut (Fig. 5). Further representative images for each of the reasons for discrepancy listed in Table 5 are given in Appendix 4.

**Table 5:** Reasons and frequency of discrepancy between human and AI reading with difference of 2 or more grades or disagreement about inadequateness in 75 images

	Reasons for discrepancy in human versus AI classification	Frequency (*n*)	Percentage (%)
1	Error in identification of the M. Pectoralis (MLO)	17	23%
2	Error in PNL measurement (CC and/or MLO)	19	25%
3	Uncertainty about correct identification of the nipples (CC and/or MLO)	19	25%
4	Error in recognizing out-of-profile nipple (CC and/or MLO)	3	4%
5	Error in categorizing nipple orientation when breast is rotated (CC)	12	16%
6	Error in capturing insufficient breast tissue (MLO)	6	8%
7	Unable to recognize skinfolds (CC and/or MLO)	9	12%
8	Dominance of a single category on overall score	4	5%
9	Unclear rationale of software scoring	8	11%

## Discussion

Currently, only a few providers have AI software solutions for dedicated quality assessment of positioning in mammography available [12]. Scientific evaluation, especially about the use in demands of clinical routine, is limited and based on rather small case collections [7], single reader comparisons [2], and tends to include only a fraction of all quality aspects. A study on the improvement of image quality and reduction of technical recalls after implementation of a direct feedback software shows promising results [14], but lacks an evaluation of the software’s performance on quality assessment criteria. Similar studies in a comprehensive setting and those investigating software validation appear to be under development.

The analysis included a fully comprehensive PGMI evaluation with all standard criteria applied and was based on multicentre real-world data in combination with reference from multireader expertise.

When comparing the human reference with the AI algorithm, the highest agreement was found for “M. pectoralis visibility” in CC and for “Pectoralis angle” in MLO. All other PGMI categories demonstrated lower levels of agreement, with some categories showing a pronounced decrease. For the overall PGMI, agreement remained consistently within the moderate spectrum.

There was low agreement on assigning an image as “inadequate.” Most of these cases had a difference of only one PGMI level. Clear delineation of inadequate images is important because of its impact on the routine screening workflow. A rating of “inadequate” may result in the exam being repeated or, if the woman has already left, in her having to return for it. It also puts a strain on the required average performance level of a team member (inadequates must be less than 3%). Software should therefore aim to reliably identify inadequate images.

For some categories, the results in the non-challenging group showed better agreement than in the challenging group of cases. In complex situations, humans may tend to interpret findings instinctively, connecting all images of a case, which can increase subjectivity. The AI, on the other hand, strictly applies to the entered rules and always takes one image at a time without establishing any relationships. Complex cases seem advanced to analyze for AI too, because either many difficulties and deficiencies occur simultaneously or reliable landmarks disappear (for example, miss of orientation when nipples are not in profile and at the same time the pectoralis is much too short in MLO) or cannot be recognized. For instance, identification of the nipple may be affected by its inconspicuous anatomical dimension, obscuration by dense glandular tissue, or preset image processing that favors ideal representation of other densities. Although the software used fully DICOM images, seemingly arbitrary values could arise if the nipple deviated significantly from the expected constellation (dimension, visibility, in profile). Also, human readers had to window extensively to recognize subtle nipples associated with high breast density. As a solution, availability to postprocessing in the graphical output of the software may avoid loss of information in the displayed image and to use the full dynamic range of the raw or DICOM images [13]. To the best of our knowledge, none of the vendors is currently able to display such extensive data within the user interface. Enabled windowing would facilitate the visualization of multilayered structures and lay the groundwork for correcting misidentified landmarks in the next step.

In PGMI, lack of the nipple in profile and the nipple within the tissue is to be classified as “inadequate,” but a nipple visualized at the skin boundary may be classified as “moderate.” This distinction worked very well for humans, but was not available for the AI system. In CC, when the nipple deviated laterally at the same time as the lateral gland body was cut, the breast might not have been sufficiently mobilized forward [3]. If the PGMI is replicated with the AI system, such a combination should be rated as “inadequate.” An explanation for the weak agreement for this measurement could be the moderate agreement for “Nipple orientation” and poor agreement for “Lateral gland.” The inappropriate result was therefore composed of two different aspects.

In a worse case, the AI system may not only process the two categories “Nipple in profile” and “Nipple orientation” (in CC) incorrectly, but also refer to an incorrect landmark for calculating the PNL. When comparing the PNL between CC and MLO, incorrect values would occur, and the initial misjudgement would affect the estimation of the other projection. In contrast, human reading may be able to instinctively establish connections and gain an understanding of the anatomical situation by considering all four projections of one woman together. We assume that AI systems would benefit from recognition of structures over different projections. In the software studied, the ipsilateral views were co-processed, allowing for evaluation of criteria such as PNL difference. Incidentally, no provider is currently able to include previous exams or other patient-related information. This would be a game changer, especially for lesion detection tools or prognostic models, and is the subject of further research [19].

In CC, the presence of M. Pectoralis was well recognized by the AI system. However, in MLO, the AI system struggled to define the outline in length and shape. Causes may include differences in anatomy, differences in positioning and differences in the contrast behavior between vendors. Furthermore, this can result in an incorrect basis for the PNL measurement. The expert radiographers review identified cases where the PNL was inconclusive for comparison, and it was not possible to ascertain which landmarks the AI utilized.

The MLO view exhibited a problematic combination that may not be adequately resolved through enhanced detection and rule adjustment. AI failure appeared to occur when part of the glandular tissue was not imaged; at the same time, the M. Pectoralis was shorter than required, and the IMF was not visualized. This situation might limit the number of landmarks to process the image correctly for the AI system, and measuring the PNL will be troublesome. One solution could be a new criterion, “Parenchyma depiction in MLO,” similar to the criteria in CC [12]. However, these criteria had poor agreement between the human reference and AI in our study.

“IMF visibility” had medium agreement with the human reference. However, it is questionable how the IMF classification can work completely without the wrinkle detection feature. This is the region where the most common unintended folds and tissue overlaps occur, due to gravity, abdominal fat, a broad-based IMF, or improper positioning along the detector edge. Since skinfolds can significantly affect image quality, it seems essential to include this criterion in the software [3].

In some cases, agreement between AI and the human reference was given in the overall PGMI score, but the individual values of the categories differed completely from each other. When the AI system produces questionable results, it would be beneficial to visually inspect the software’s pattern recognition and underlying calculations to gain a deeper understanding of its functionality and the rationale behind its outputs. A graphical representation of the registered structures and lines within the interface, and human adaptation options seem inevitable [2, 12]. A future goal may be to design a practical platform that enables adjustment and communication between the user and the software, as is already possible with one vendor [20].

The variability between the model output and a manual PGMI assessment can generally also be attributed to differences in the evaluation methodology. While human reading remains prone to subjective tendencies, the evaluation by the algorithm consists of explicitly applying sets of rules relying on explicit measurements.

In fact, replication of traditional PGMI by an AI algorithm is difficult and not yet sufficient for a direct transfer into routine. Nevertheless, we have been able to obtain information about which aspects of the current PGMI are easier to transfer to an artificial model and which are more troublesome. This raises the question of whether a one-to-one transfer of the PGMI, with its human interpretations at the limits of objectivity, to a technically structured AI can work despite deep learning and similar approaches, or whether our criteria and methods should first be re-evaluated and discussed in the light of new possibilities. In any case, transparent feedback to programmers and designers of software can be a relevant contribution to finding an appropriate quality assessment for the future.

### Limitations

Although the study sample included 800 images from 13 different sites, a larger number of cases would have given more power to the study. When selecting the participating institutions, great importance was attached to a range in terms of unit size, throughput, team constellation, and clientele to allow a heterogeneity of the data that corresponds to reality. We wanted to ensure that the concept would work equally well in different environments and not just in strictly organized screening scenarios with highly experienced core teams and fast workflows. Precisely because of this, the distribution of P, G, M, and I, as well as the distribution of the individual flaws, was not homogeneous in our approach. Further investigation would benefit from a pre-selected and balanced set of data to lay the groundwork for validation at a later stage.

The software recorded fewer PGMI criteria than the human readers in their familiar system (Appendix 1). It did not detect skinfolds or blur/motion artifacts, which may have affected the agreement between the overall PGMI score. However, when assessing the IMF, the missing skinfold category may have had less influence on the evaluation, since the presence of overlap effects in this region could already be detected by the software upon specific request to the provider. For a 1:1 comparison, the humans would have to re-evaluate using a new table or wait for further expansion of the missing categories.

All readers strictly recorded every single criterion of each image. The overall PGMI score they determined for each image was moreover based on their own subjective assessment. This means that weighting comes into play in the overall PGMI value. The participants were familiar with this procedure from their respective systems. Since we wanted results that would also be produced in routine, we left this approach as it was. This subjective impact poses a significant challenge for computer software, which is obviously better suited to replicating rules and standards. The AI followed the concept of the lowest individual value as a grade for the overall PGMI per image. Other more balanced approaches, adding more weight to specific PGMI categories, would have been more conclusive to compare with the human reading classifications, but this was not available in the prototype of the AI system. Recognizing this discrepancy, our study compared not only the overall PGMI but also the values of each individual criterion.

The PGMI assessment by the software was primarily based on structural recognition and rule-based categorization, rather than advanced learning from a broad base of human evaluations. The inclusion of additional data and training on a larger scale is considered essential in order to advance the software and ultimately achieve reliable validation and convincing establishment on the market.

The study did not focus on interreader agreement, as data have already been published [3, 6, 9, 13, 21]. We were not able to conduct a large multinational, multireader study; however, the human expert consensus reading in our study may provide a PGMI reference standard.

To avoid any bias in a consensus voting or re-reading process, we recommend using specially designed software with complete blinding capabilities whenever possible.

A larger consensus on requirements across screening programs and organizations may be fruitful to harmonize quality assessment in mammography and ultimately define the framework for AI.

### Conclusion

The results from this study showed that the transformation of mammographic image quality measurements using PGMI into a fully automated AI system is challenging. Although moderate agreement was reached for overall PGMI, performance across specific criteria was inconsistent and decreased substantially in challenging images. Elaboration of the decision-making process and criteria for human assessment of mammographic image quality is essential in further work with automated solutions aimed at replacing and supporting radiographers as an objective, time- and cost-effective tool.

## Supplementary information

Supplementary Material

## Data Availability

All mammography image data were collected in the period from June to August 2021 and are courtesy of the University Hospital of Innsbruck, Austria, or the Hirslanden Group, Switzerland. The CRFs with the evaluated PGMI data are archived by T.S.

## Abbreviations

AI: Artificial intelligence
CC: Craniocaudal
CI: Confidence interval
IMF: Inframammary fold
MAE: Mean absolute errors
MLO: Mediolateral oblique
PGMI: Perfect-good-moderate-inadequate
PNL: Pectoralis nipple line
