# Supplementary material for: AI-based image quality assessment of positioning in mammography: considerations and challenges
Source: Insights Imaging. 2026 Feb 16;17:47. doi: 10.1186/s13244-025-02191-3 (PMC12909628; doi:10.1186/s13244-025-02191-3)
Supplement: Supplementary file 1 — Supplementary Material [file 13244_2025_2191_MOESM1_ESM.pdf]

AI-based image quality assessment of positioning in mammography: considerations and challenges

ELECTRONIC SUPPLEMENTARY MATERIAL

Appendix 1.

PGMI criteria (study version)

**CC**				
**criteria**	**P**	**G**	**M**	**I**
technical image quality (overexposure, underexposure, contrast, resolution, sharpness, noise, artifacts	none	yes, but not in relevant areas, unrestricted diagnostics	yes, somewhat restrictive	massive, problematic for diagnostics
visually adequate compression	appears as adequate	appears as reduced	appears as too less	insufficient compression
motion artifacts	none	yes, but not in relevant areas, unrestricted diagnostics	yes, somewhat restrictive	massive, problematic for diagnostics
non-technical artifacts (body parts, contralateral breast, external objects, other)	none	yes, but not in relevant areas, unrestricted diagnostics	yes, somewhat restrictive, but not over glandular tissue	massive, artifacts over glandular tissue, problematic for diagnostics
symmetry	accurate	nearly	disturbing<br>(e.g. just on the edge)	massive, problematic for diagnostics<br>(e.g. cut)
pectoral muscle visibility	visible	not visible	-	-
PNL length	PNL CC = PNL MLO (difference <1mm)	slightly shorter (difference <5mm)	PNL CC shorter but difference <15mm	PNL CC shorter and difference >15mm
PNL length (distance in mm)				
nipple profile	in profile	partially in profile	not in profile<br>(nipple touches the skin border)	not in profile<br>(projected into the tissue)
nipple orientation	straight	<20° to medial	<20° to lateral	>20° to medial or lateral
medial gland	complete	slightly complete	parts missing although nipple straight	larger parts missing and medial nipple
lateral gland	complete	slightly complete	parts missing although nipple straight	larger parts missing and lateral nipple
skinfolds	none	small wrinkles, but not in relevant areas, unrestricted diagnostics (windowing possible)	folds, but outside of glandular tissue (overlapping folds, no windowing possible)	massive folds, strongly overlapping folds, folds over glandular tissue, problematic for diagnostics
skinfolds localization (lateral, medial, pectoral, others)				
visible scar (yes/no)				
visible lesion (yes/no)				
**Summary PGMI value**				

**MLO**				
**criteria**	**P**	**G**	**M**	**I**
technical image quality (overexposure, underexposure, contrast, resolution, sharpness, noise, artifacts	none	yes, but not in relevant areas, unrestricted diagnostics	yes, somewhat restrictive	massive, problematic for diagnostics
visually adequate compression	appears as adequate	appears as reduced	appears as too less	insufficient compression
motion artifacts	none	yes, but not in relevant areas, unrestricted diagnostics	yes, somewhat restrictive	massive, problematic for diagnostics
non-technical artifacts (body parts, contralateral breast, external objects, other)	none	yes, but not in relevant areas, unrestricted diagnostics	yes, somewhat restrictive, but not over glandular tissue	massive, artifacts over glandular tissue, problematic for diagnostics
symmetry	accurate	nearly	disturbing<br>(e.g. just on the edge)	massive, problematic for diagnostics<br>(e.g. cut)
pectoralis muscle relaxation and length	relaxed and to nipple level	not relaxed and/or to PNL level	not to PNL level	not or only slightly visible
pectoral angle	≥20°	≥10°	<10°	not or only slightly visible
PNL	PNL MLO = PNL CC (difference <1mm)	slightly shorter (difference <5mm)	PNL MLO shorter but difference <15mm	PNL MLO shorter and difference >15mm
PNL length (distance in mm)				
nipple profile	in profile	partially in profile	not in profile<br>(nipple touches the skin border)	not in profile<br>(projected into the tissue)
IMF visibility	visible and plain	slightly visible or small wrinkles	not visible	caudal tissue missing

skin folds	none	small wrinkles, but not in relevant areas, unrestricted diagnostics (windowing possible	folds outside of glandular tissue (overlapping folds, no windowing possible)	massive folds, folds over glandular tissue, problematic for diagnostics
skin folds localization (IMF, pectoral, cranial, others)				
visible scar (yes/no)				
visible lesion (yes/no)				
**Summary PGMI value**				

Overview of assessed and analyzed PGMI criteria

PGMI criteria	Assessed by human readers	Assessed by software	Included in statistical analysis
**CC+MLO criteria**			
technical image quality (overexposure, underexposure, contrast, resolution, sharpness, noise, artifacts	✓	✓	
visually adequate compression	✓		
motion artifacts	✓		
non-technical artifacts (body parts, contralateral breast, external objects, other)	✓		
symmetry	✓	✓	
**CC only criteria**			
pectoral muscle visibility	✓	✓	✓
PNL length	✓	✓	✓
PNL length (distance in mm)	✓	✓	
nipple profile	✓	✓	✓
nipple orientation	✓	✓	✓
medial gland	✓	✓	✓
lateral gland	✓	✓	✓
skinfolds	✓		
skinfolds localization (lateral, medial, pectoral, others)	✓		
visible scar (yes/no)	✓		
visible lesion (yes/no)	✓		
**CC Summary PGMI value**	✓	✓	✓
**MLO only criteria**			
pectoralis muscle relaxation and length	✓	✓	✓
pectoral angle	✓	✓	✓
PNL	✓	✓	✓
PNL length (distance in mm)	✓	✓	
nipple profile	✓	✓	✓
IMF visibility	✓	✓	✓
skin folds	✓		
skin folds localization (IMF, pectoral, cranial, others)	✓		
visible scar (yes/no)	✓		
visible lesion (yes/no)	✓		
**MLO Summary PGMI value**	✓	✓	✓

Appendix 2.

Examples of the visual output of the software

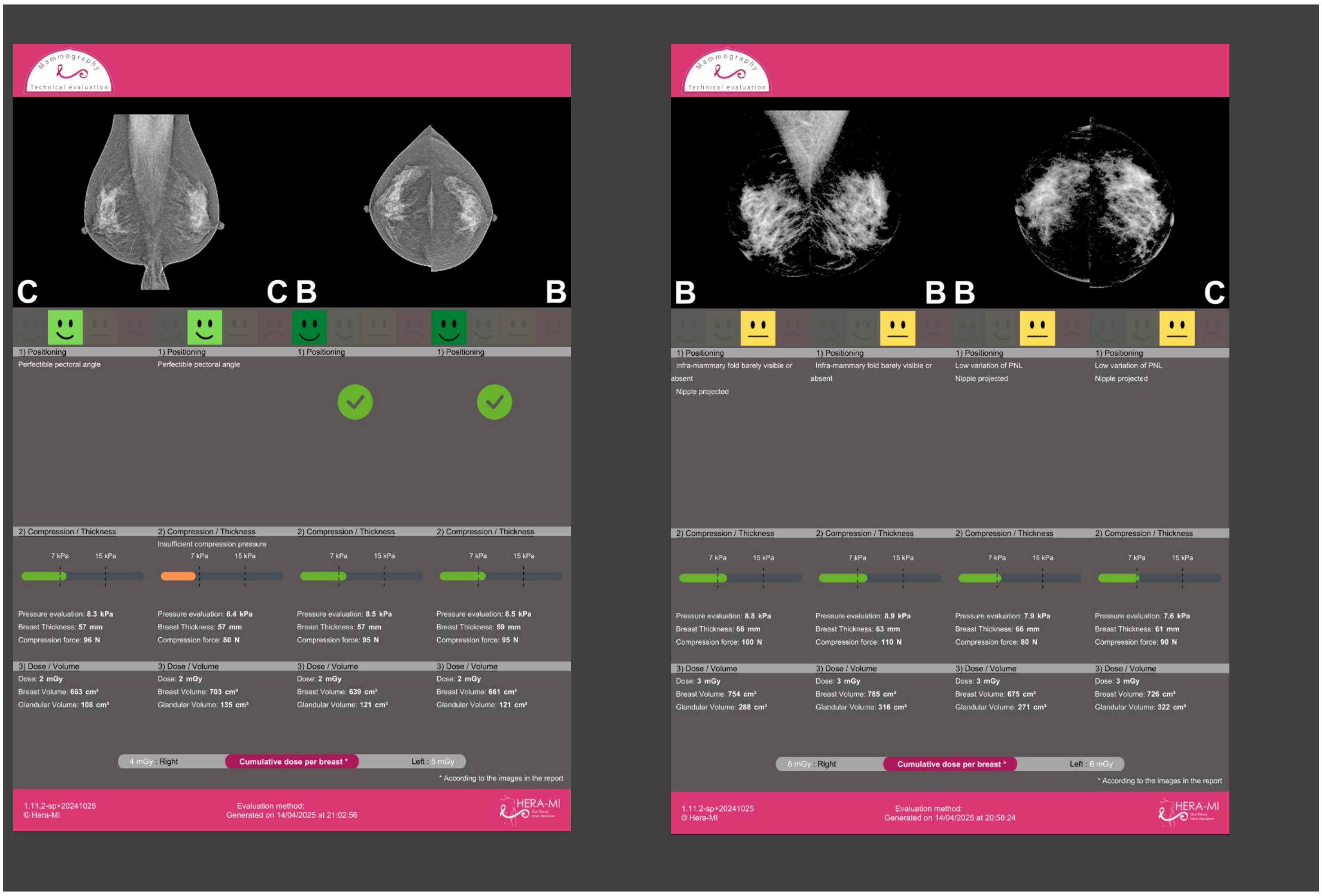

Appendix 3.

Differences in the PGMI (Perfect, Good, Moderate, Inadequate) scoring between the human readers and the AI system. A difference of 1 could for example mean that AI scored an image as P and human readers as G, or human readers as M and AI as I. If the difference was 3, AI had scored P and human readers I, or the other way around. Error/NA referred to no consensus possible or too little information on the image to give a fair grade. Results are stratified by non-challenging and challenging cases. Results for all cases are given in Table 2.

**Craniocaudal images**	Non-challenging cases (n=260)										Challenging cases (n=140)									
	Agreement		Difference=1		Difference=2		Difference=3		Error/NA		Agreement		Difference=1		Difference=2		Difference=3		Error/NA	
M. Pectoralis visibility	225	87%	30	12%	0	0%	0	0%	5	2%	126	90%	13	9%	0	0%	0	0%	1	1%
PNL comparison	118	45%	102	39%	25	10%	0	0%	15	6%	47	34%	47	34%	34	24%	3	2%	9	6%
Nipple in profile	166	64%	81	31%	10	4%	0	0%	3	1%	16	11%	84	60%	35	25%	0	0%	5	4%
Nipple orientation	157	60%	86	33%	9	3%	0	0%	8	3%	69	49%	46	33%	20	14%	0	0%	5	4%
Medial gland depiction	103	40%	95	37%	37	14%	12	5%	13	5%	46	33%	61	44%	19	14%	4	3%	10	7%
Lateral gland depiction	69	27%	81	31%	76	29%	17	7%	17	7%	16	11%	52	37%	37	26%	26	19%	9	6%
Overall PGMI	123	24%	124	24%	9	2%	0	0%	4	1%	62	22%	68	24%	6	2%	0	0%	4	1%
**Mediolateral Oblique Images**	Non-challenging cases (n=260)										Challenging cases (n=140)									
	Agreement		Difference=1		Difference=2		Difference=3		Error/NA		Agreement		Difference=1		Difference=2		Difference=3		Error/NA	
M.Pectoralis relaxation and length	91	35%	108	42%	39	15%	15	6%	7	3%	35	25%	46	33%	32	23%	24	17%	3	2%
Pectoralis angle	191	73%	59	23%	6	2%	3	1%	1	0%	87	62%	43	31%	3	2%	3	2%	4	3%
PNL comparison	167	64%	76	29%	9	3%	0	0%	8	3%	54	39%	64	46%	12	9%	2	1%	8	6%
Nipple in profile	159	61%	84	32%	7	3%	0	0%	10	4%	13	9%	60	43%	62	44%	0	0%	5	4%
IMF visibility	134	52%	109	42%	16	6%	1	0%	0	0%	57	41%	51	36%	23	16%	1	1%	8	6%
Overall PGMI	141	27%	105	20%	13	3%	1	0%	0	0%	77	28%	55	20%	7	3%	0	0%	1	0%

Appendix 4.

Image examples for each of the reasons for discrepancy

1. Error in identification of the M. pectoralis (MLO)

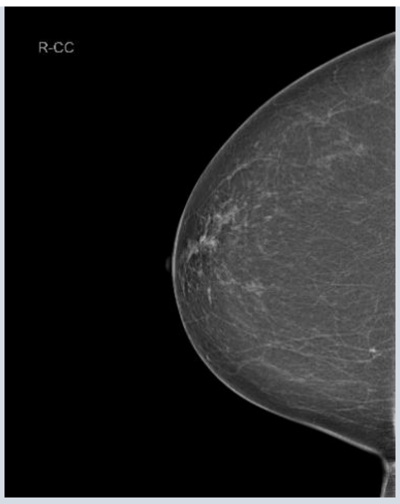

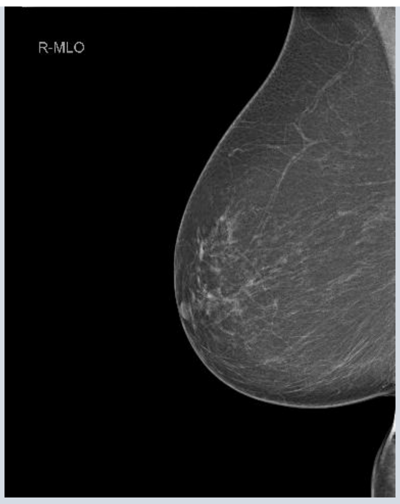

Case 20 – RMLO

Human reader consensus: inadequate

Pectoralislength=moderate

PNL=inadequate

(128.3mm RMLO vs. 133.1mm RCC, estimated true PNL ~148mm)

IMF=moderate

AI: moderate

Pectoralislength=perfect

PNL=good

(77.90mm RMLO vs. 79.09mm RCC)

IMF=moderate

→

wrong pectoralis detection leads to wrong estimation of the PNL

2. Error in PNL measurement (CC and MLO)

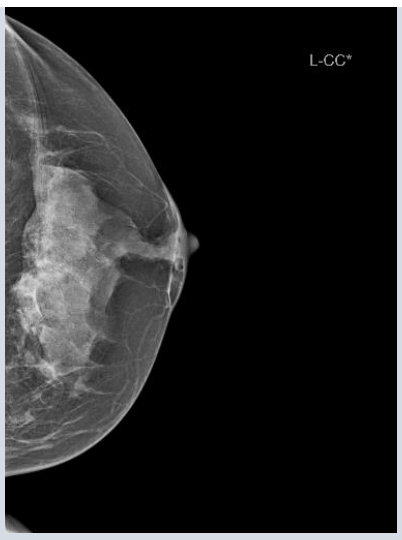

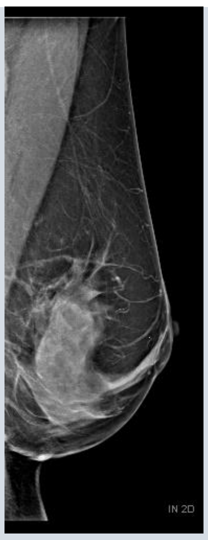

Case 77 – LCC

Human reader consensus: inadequate

PNL=inadequate

(79.6mm LCC vs. 96.9mm LMLO)

Nippleprofile=perfect

Nippleorientation=perfect

Medial gland=moderate

Lateral gland=good

AI: good

PNL=perfect

(61.18mm LCC vs. 58.16mm LMLO)

Nippleprofile=good

Nippleorientation=perfect

Medial gland=perfect

Lateral gland=perfect

→

incomprehensible PNL measurement

3. Uncertainty about correct identification of the nipples (CC and MLO)

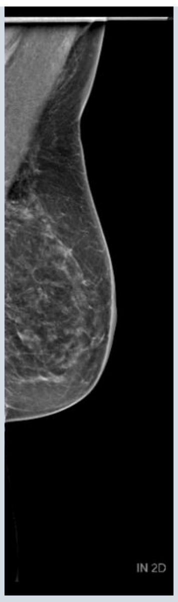	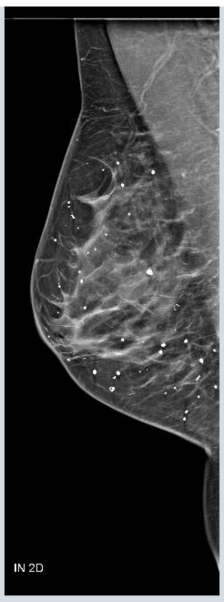 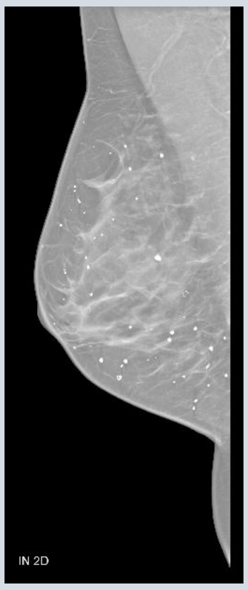	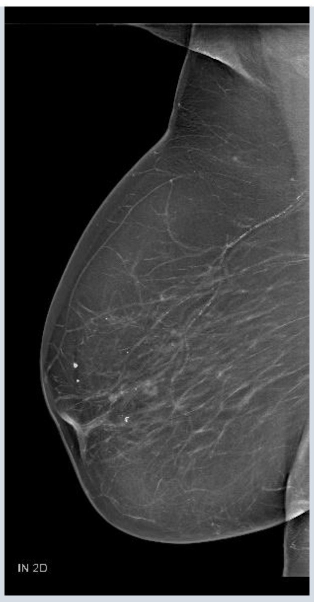
Case 54 – LMLO<br><br>Nipple not visible	Case 110 – RMLO<br><br>Nipple hardly visible without windowing<br>(original image left, adapted image right)	Case 56 – RMLO<br><br>Anatomically retracted nipple

4. Error in recognizing out of profile nipple (CC and MLO)

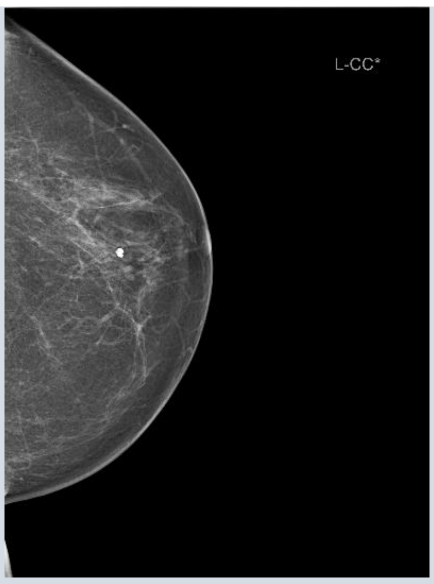	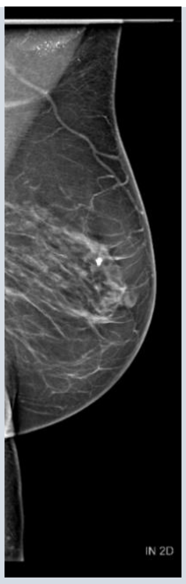	Case 55 – LMLO<br><br>*Human reader consensus: inadequate*<br>Pectoralislength=moderate<br>PNL=moderate<br>**Nipple=inadequate**<br>IMF=moderate<br>Comment: glandular tissue missing, rotated breast<br><br>*AI: moderate*<br>Pectoralislength=perfect<br>PNL=good<br>**Nipple=moderate**<br>IMF=good<br><br>→<br>distinction between moderate and inadequate out of profile nipple

5. Error in categorizing nipple orientation when breast is rotated (CC)

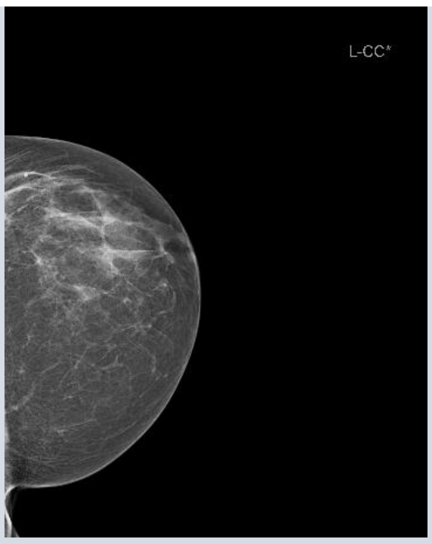	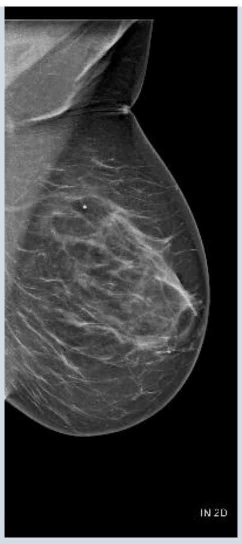	Case 6 – LCC<br><br>*Human reader consensus: inadequate*<br>PNL=moderate<br>**Nippleorientation=inadequate**<br>**Lateral gland=inadequate**<br><br>AI: good<br>PNL=good<br>**Nippleorientation=good**<br>**Lateral gland=perfect**<br><br>→<br>deviation of nipple orientation plus glandular tissue cut due to rotated breast position

6. Error in capturing insufficient breast tissue (MLO)

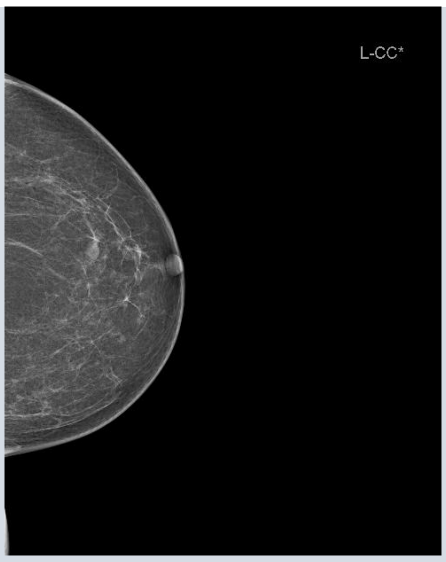	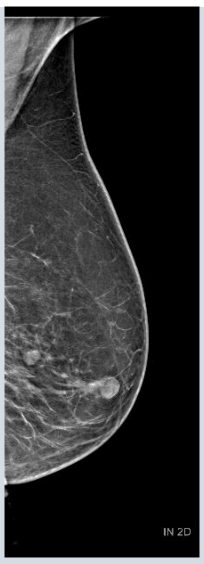	Case 23 – LMLO<br><br>*Human reader consensus: inadequate*<br>**Pectoralislength=inadequate**<br>**PNL=inadequate**<br>(57.6mm LMLO vs. 87.5mm LCC,<br>estimated true PNL ~76mm)<br>Nipple=inadequate<br>**IMF=inadequate**<br>Comment: large parts of breast missing<br><br>*AI: moderate*<br>**Pectoralislength=perfect**<br>**PNL=perfect**<br>(72.34mm LMLO vs. 54.43mm LCC)<br>Nipple=moderate<br>**IMF=good**<br><br>→<br>error in detection of multiple deficiencies, possibly too few landmarks

7. Unable to recognize skinfolds (CC and MLO)

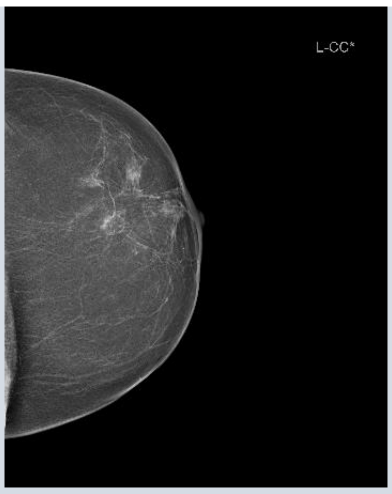

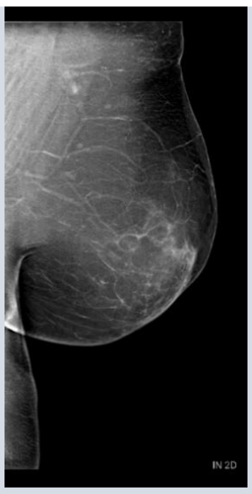

Case 117 – LMLO

Human reader consensus: inadequate

Nipple=moderate

IMF=moderate

Skinfolds=inadequate

AI: moderate

Nipple=moderate

IMF=good

Skinfolds=n.a.

→

massive fold in IMF, limited diagnostic accuracy in lower dorsal area

8. Dominance of a single category on overall score

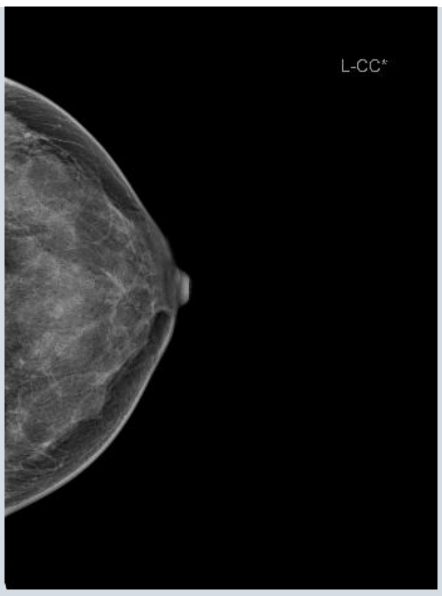

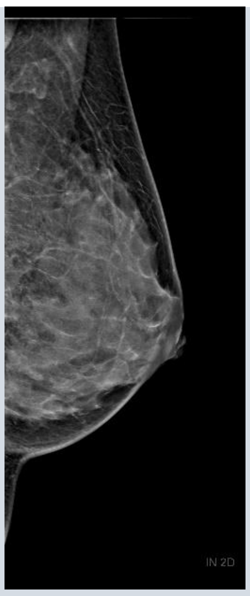

Case 82 – LMLO

Human reader consensus: perfect

Pectoralislength=perfect

Pectoralisangle=perfect

PNL=perfect

Nipple=good

IMF=perfect

AI: moderate

Pectoralislength=perfect

Pectoralisangle=good

PNL=good

Nipple=moderate

IMF=perfect

→

awarding a moderate just because of a partially profiled nipple is not justifiable for the human readers

9. Unclear rationale of software scoring

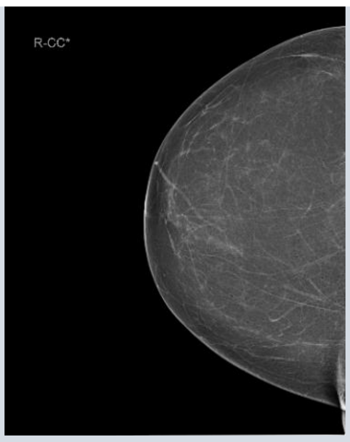	Case 66 - RCC<br><br>*Human reader consensus: inadequate*<br>PNL=inadequate (PNL 136.3mm)<br>Nipple: not clearly visible<br>Comment: none of the clips are visible<br><br>*AI: moderate*<br>PNL=moderate (79.68mm)<br>Nippleprofile=good<br>Nippleorientation=good
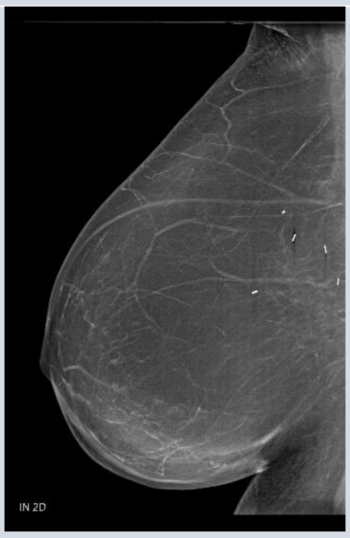	Case 66 – RMLO<br><br>*Human reader consensus: moderate*<br>**Pectoralislength=moderate**<br>**Pectoralisangle=moderate**<br>PNL=good<br>(179.2mm, estimated true length ~184mm)<br>Nipple=perfect<br>IMF=perfect<br><br>*AI: moderate*<br>Pectoralislength=good<br>Pectoralisangle=perfect<br>PNL=perfect<br>**Nipple=moderate**<br>**IMF=moderate**<br>(103.54mm)<br><br>→<br>agreement in overall PGMI score for MLO with different criticized single criterions
